# A Green Electromagnetic Energy Harvester with Up-Frequency and Unidirectional Rotation for Smart Pavement

**DOI:** 10.3390/ma18040786

**Published:** 2025-02-11

**Authors:** Keliang Mou, Xiaoping Ji, Xiaojuan Li, Haoyu Zhou, Yunrui Wu, Yeyang Fang

**Affiliations:** 1 School of Highway, Chang’an University, Xi’an 710064, Chinaarmstrongzhou@foxmail.com (H.Z.); 13640304916@163.com (Y.W.); 15268503615@163.com (Y.F.); 2 Technology Development Group Co., Ltd., Shaanxi Transportation Holding Group, Xi’an 710075, China; lxiaojuan2024@163.com

**Keywords:** smart pavement, up-frequency, unidirectional rotation, electromagnetic energy harvester, electrical output

## Abstract

Smart pavement is composed of a monitor network, communication network, data center, and energy supply system, and it requires reliable and efficient energy sources to power sensors and devices. The mechanical energy is wasted and dissipated as heat in traditional pavement; this energy can be reused to power low-power devices and sensors for smart pavement. Mechanical energy harvesting systems typically perform through electromagnetic, piezoelectric, and triboelectric methods. Among the different methods, electromagnetic harvesters stand out for their higher output power. However, current electromagnetic harvesters face challenges such as bulky designs, low power density, and high input displacement requirements. This study proposed a green electromagnetic harvester (GEH) based on up-frequency and a unidirectional rotation mechanism to harvest mechanical energy from the pavement. A prototype was designed and prepared. The influence of different parameters on the electrical performance of the harvester was studied by using an MTS test instrument and simulation methods. The results demonstrate that increasing the frequency and optimizing the magnetic array significantly enhances electrical output. The open-circuit voltage in the N-S mode is 3.1 times higher than that in the N-N mode. At a frequency of 9 Hz and a displacement of 3.0 mm, the open-circuit voltage of the GEH is 6.73 V, the maximum power output is 171.14 mW, the peak power density is 1277.16 W/m^3^, and the voltage has almost no decay after 100,000 cycles. Further, the application of the GEH in charging sensors and capacitors was demonstrated, which indicates the potential of a GEH to power sensors for smart roads.

## 1. Introduction

With the rapid development of the Internet of Things, intelligent demands are increasingly essential in the transportation industry. The widespread deployment of intelligent sensors across road domains can lay the foundation for real-time road information sensing [1]. However, the continuous supply of energy for these sensors remains a challenge for their large-scale application. Using batteries as a power source presents issues such as difficulty of regular maintenance and replacement, as well as the environmental pollution caused by battery disposal. As a universal infrastructure, roads generate considerable renewable mechanical energy due to frequent traffic during operation [2]. Converting mechanical energy into electrical energy through energy harvesting technology can continuously power road sensors, reduce road energy consumption and promote the advancement of smart roads. Generally, there are three methods to convert mechanical energy into electrical energy by using energy harvesting technology in roads: piezoelectric energy harvester (PEH), triboelectric nanogenerator (TENG), and electromagnetic harvester (EMH) [3,4,5].

PEHs utilize the piezoelectric effect to transform mechanical energy into electrical energy, and numerous studies on road piezoelectric energy harvesters have been reported [6]. Wang et al. designed a cantilever beam structure suitable for harvesting energy from low-frequency road vibrations. The determined size of the cantilever beam structure was optimized by simulation. Under experimental conditions of 12 Hz frequency and 1.0 mm displacement, the PEH produced an output voltage of 24 V and an output power of 4.38 mW [7]. Cao et al. prepared a PEH to produce 5.8 mW of output power under external stimulation of 15 Hz–0.7 MPa. After 500,000 cycles of loading, the voltage decreased by only 2%, indicating that the PEH has good long-term stability [8,9]. Jasim et al. designed a bridge energy harvester with layer polarization. Under the load of 0.7 MPa, the potential energy generated by a single PEH was 0.743 mJ [10]. In addition to the aforementioned designs, other PEHs such as stacked [11], drum [12], cymbal [13], and flexible [14] have also been reported. The above literature fully confirms the viability of the road piezoelectric energy harvesting technology. Furthermore, the practical application of the energy harvesters on the road has been explored. Xiong et al. installed a square PEH harvester at a highway weighing station in the U.S., where it produced an average output power of 0.116 W from vehicles with different axle loads passing by [15]. Hong et al. installed an open piezoelectric device on the pavement, which powered a wireless temperature sensor monitoring system and a cell phone battery [16]. Nevertheless, piezoelectric materials exhibit large internal resistance and small output current, which are not conducive to the effective storage of electric energy, and require a larger volume to improve the output performance.

TENG is designed based on the coupling effect of triboelectrification and electrostatic induction; it is another essential type of mechanical energy harvesting [17]. Heo et al. developed a TENG speed bump. When vehicle tires drive over the PVC speed bump, the energy generated by the harvester lights up 120 LED lights [18]. Zhu et al. designed an origami-structure triboelectric nanogenerator with a current output of 3.61 μA at a frequency of 18 Hz [5]. Although the TENG is simple to prepare and easy to integrate, its materials are easily damaged by friction [19].

The energy conversion principle of EMH is based on Faraday’s law. In typical electromagnetic mode, a vertical displacement is applied to the harvester, causing the coil and magnet to move relative to each other, thereby generating an induced current in the coil. To achieve this conversion mechanism in the road, some mechanical systems such as rack–pinion, hydraulic, and cam-arms assist jointly in driving the harvester to work [20]. For example, Gholikhani et al. prepared an EMH containing a rack–pinion. The results show that the average output power of the harvester was 3.21 mW [21]. Subsequently, Gholikhani et al. designed a hybrid EMH. The harvester consists of two mechanisms, a linear generator and a rack and pinion. At 13 mm displacement, the actual output square root power of the harvester was 1.2 W and 80 mW [22]. Zabihi et al. prepared an EMH that mainly uses crank to achieve motion conversion, with four spring supports at the plate corners for component reset [23]. The test shows that the average power output of the energy harvester reaches 2.24 W (18 mm displacement) and 1.5 W (25 mm displacement), respectively. Similarly, Bano et al. prepared an EMH applied in high-speed toll stations [24]. The motion conversion of the harvester involves transferring the vertical motion of the piston through the crank mechanism and converting it into the rotating motion of the gear. The maximum output voltage of the energy harvester is 6.9 V at a speed of 5 mm/s. Li et al. designed a hydraulically driven rotating electromagnetic energy harvester that can be installed in road speed bumps with a maximum output power of 0.35 W [25]. However, excessive vertical downward displacement can significantly affect driving comfort and safety.

To further reduce vertical displacement, Liu et al. designed a unidirectional rotating EMH using a rack, pinion, one-way clutch, and flywheel to harvest energy from human walking. At a displacement of 6.0 mm, the average harvested energy was 1.8 J [26]. Sun et al. proposed a harvester that uses a double V-shaped crank mechanism to transform vertical linear motion into a unidirectional rotating motion [27]. Under conditions of 500 N and 0.1 Hz, the harvester achieves a peak voltage of 12.64 V and an output power of 1.1 W. In addition to vertical displacement motion transformation, Jiang et al. proposed a sliding-plate-based transverse deceleration EMH for road tunnels [28]. At a 1.5 Hz frequency and a horizontal amplitude 10 mm, the average output power of the harvester is 5.135 W.

EMH harvesting sidewalk energy is also gaining attention. Cao et al. prepared a double rocker EMH to collect human footstep energy [1]. The maximum output power of 466.6 mW was obtained under the condition of vibration frequency of 5 Hz and displacement of 10 mm. Asadi et al. adopted up-frequency technology to transform the gravity exerted by pedestrians into vibrational motion, and further simulated and optimized the arrangement of magnets [29]. Zou et al. proposed a bidirectional EMH with a slow-release regulation mechanism, and the maximum voltage and total electric energy generated by a pedestrian walking at a speed of about 6 km/h were 51.2 V and 5.4 J, respectively [30].

Road energy harvesters based on the electromagnetic effect provide a viable method for road mechanical energy conversion. However, there are still some problems with current EMHs. First, the design involves large vertical displacement, which may affect driving comfort; second, the use of various mechanical components to achieve vertical displacement conversion not only increases the size of the energy harvester and reduces power density and conversion efficiency, but also makes it prone to failure due to complex component coordination.

This paper proposes an energy harvester with up-frequency and unidirectional rotation for road mechanical energy harvesting and utilization. By limiting the displacement, the low-frequency excitation of the road is transformed into unidirectional rotation and up-frequency, and the conversion of kinetic energy into electrical energy according to Faraday’s law improves the efficiency of energy collection and utilization. The performance of the designed structure is optimized and evaluated through finite element analysis and experiments. Finally, the practicability of GEH is verified by charging the device, which lays the foundation for the green and low-carbon power supply of smart roads.

## 2. Design and Working Mechanism of the GEH

### 2.1. Structural Design Overview

**Figure 1:**
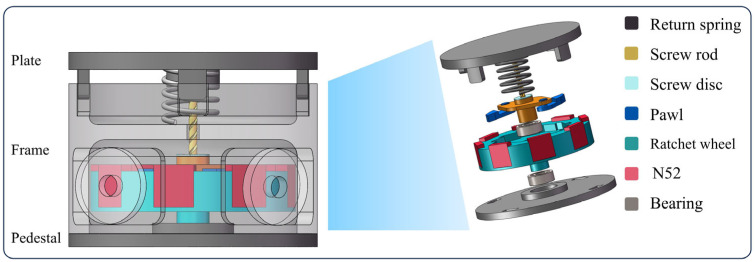
The design of the green electromagnetic harvester.

The overall structure of a GEH primarily contains a motion conversion module, an up-frequency and unidirectional rotation module, and an electromechanical conversion module. The motion conversion module mainly collects the road mechanical energy and converts the vertical motion into rotary motion. It primarily comprises five parts: screw rod, return spring, screw disc, pawl, and bearing. The up-frequency unidirectional rotation module mainly consists of a ratchet wheel and a bearing, which can achieve up-frequency, rotary drive, and unidirectional rotation. The energy conversion module adopts a magnet-coil rotation system to facilitate power conversion. The prepared GEH prototype is shown in Figure 1.

The screw rod and screw disc are made of M2 high-speed steel, an alloy steel composed of carbon (C), tungsten (W), molybdenum (Mo), chromium (Cr), and cobalt (Co). The carbon content is approximately 0.85%, which enhances the hardness and wear resistance of the steel, ensuring long-term friction performance for the screw rod and screw disc. The magnet used is NdFeB-N52, with a residual magnetic induction intensity of 1.42–1.48 T and an operating temperature limit of ≤80 °C. The four coils are placed in the four slots of the frame. The coils are wound with 500 turns of enameled copper wire. According to the literature [31], the optimal coil diameter is 0.12 mm. The plate, frame, and base are made of nylon 12 material through 3D printing. The average compressive strength tested is 32.6 MPa, which can meet the compressive requirements of the road environment.

Existing electromagnetic energy harvesters suffer from excessive volume and low power density [23,26]. Considering the matching of equipment installation and asphalt pavement surface thickness (4~5 cm), the electromagnetic energy harvester with a height of no more than 5 cm is designed, further enhancing structural compactness and power density. Additionally, vehicle-induced excitation on road energy harvesters is stochastic and occurs at low frequencies. To improve the electrical performance of the electromechanical conversion module, the simple ratchet pawl structure is selected, which not only realizes the vertical direction to unidirectional rotation but also improves the low-frequency excitation.

In practical road energy harvesting scenarios, researchers just consider the energy harvest efficiency, and adopt the input displacement with a vertical height greater than 1 cm [22,23]. However, the centimeter-level deformation may affect the safety of driving and reduce energy efficiency. Typically, the height of the road marking is 1.0–2.0 mm, and the permissible maximum deformation for driving stability and comfort is generally no more than 3.0 mm [32]. Therefore, the vertical displacement of the prototype designed in this study is 3.0 mm.

### 2.2. Working Principle of the GEH

**Figure 2:**
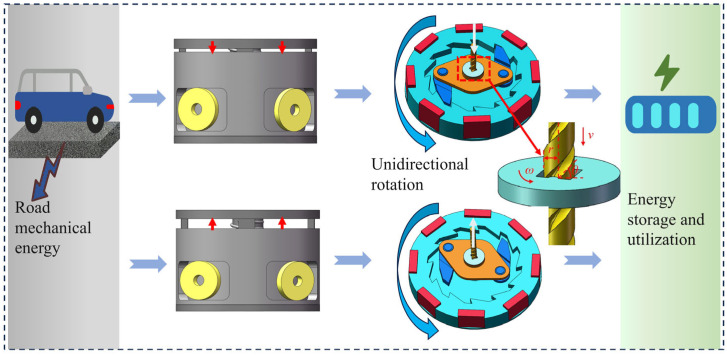
Schematic diagram of the working principle and energy harvesting of the energy harvester.

Figure 2 shows the working principle of the GEH. When the plate of the GEH is stressed by a vehicle load, the screw rod moves downward. The screw rod and screw disc engage to drive the pawl mounted on the bearing, causing it to rotate. The pawl interlocks with the ratchet wheel, driving it to rotate counterclockwise, thereby increasing its rotational speed. After the vehicle load disappears, the return spring pushes the screw rod to reset upwards and brings the pawl to reverse rotation. The pawl slides out of the ratchet wheel and the ratchet continues to turn counterclockwise. Therefore, whether the plate is up or down, the ratchet always remains in one direction, achieving a one-way rotation. Up-frequency conversion is achieved through ratchet inertia, which sustains rotation and extends the rotation duration. When the ratchet wheel rotates in a direction, the movement of the N52 magnet generates an induced voltage in the stationary coil.

When the vehicular load is applied to the top plate of the composite energy harvester, the top plate acquires a downward velocity *v*, driving the screw rod to move downward synchronously. The downward motion of the screw rod is subsequently converted into the rotational motion of the screw disc. Over a time duration *t*, the vertical displacement *h* of the torsion bar is expressed as follows:

(1) h=vt

The horizontal displacement *l* generated by the screw disc is expressed as follows:

(2) $$l=\frac{h}{\tan\theta }$$

where *θ* is the thread angle of the screw bar.

Since the screw disc and screw rod share the same displacement to prevent relative sliding at their contact interface, the angular velocity of the screw disc (*ω*) and the displacement of the screw rod (*h*) are related as follows:

(3) ωrt=l

where *r* is the effective radius of the screw disc.

Substituting *h* from Equation (1) into (2), and then substituting *l* from Equation (2) into (3), the angular velocity *ω* of the torsion disc can be expressed as follows:

(4) $$\omega =\frac{v}{r\tan\theta }$$

Since the screw disc and the pawl structure form an integrated unit, the maximum angular velocity of the pawl is equal to that of the screw disc. When the pawl drives the ratchet wheel to rotate, the axis of the coil is perpendicular to the surface of the magnet. Therefore, only the magnetic flux in the *z*-axis direction needs to be considered. According to Faraday’s law, the induced voltage *V_e_* is as follows [30]:

(5) $$V_{e}=-N\frac{d\varphi }{dt}=-N\frac{BSd\cos\omega t}{d\alpha }\cdot \frac{d\alpha }{dt}$$

where *N* is the coil number of turns, *S* is the effective area, *B* is the magnetic flux density, and *ω* and *α* are the angular velocity and phase angle of the ratchet wheel, respectively. With the coil connected in series, the output power *P* of the load is as follows:

(6) $$P={\left(\frac{V_{e}}{R_{coil}+R_{e}}\right)}^{2}R_{e}={\left(\frac{N\omega BS}{\left(R_{coil}+R_{e}\right)}\frac{d\cos\omega t}{d\alpha }\right)}^{2}R_{e}$$

where *R_e_* is the circuit resistance and *R_coil_* is the coil resistance. According to Equation (6), when *R_e_* is equal to *R_coil_*, the maximum output power of the electromagnetic output is as follows:

(7) $$P_{\max}=\frac{1}{4R_{e}}{\left(\frac{N\omega BSd\cos\omega t}{d\alpha }\right)}^{2}$$

## 3. Output Optimization of the GEH

### 3.1. Optimization of the Number and Arrangement of Magnets

The output performance of the GEH relies on the magnetic field intensity *B*, which is directly determined by the array of magnets. Varying the number and arrangement of magnets results in different magnetic field distributions. In order to investigate the magnetic field distribution of the different magnets, the finite element method was used to numerically study magnet arrays. The simulation parameter settings are shown in Table 1. All simulations were completed in COMSOL Multiphysics 6.0 software.

**Figure 3:**
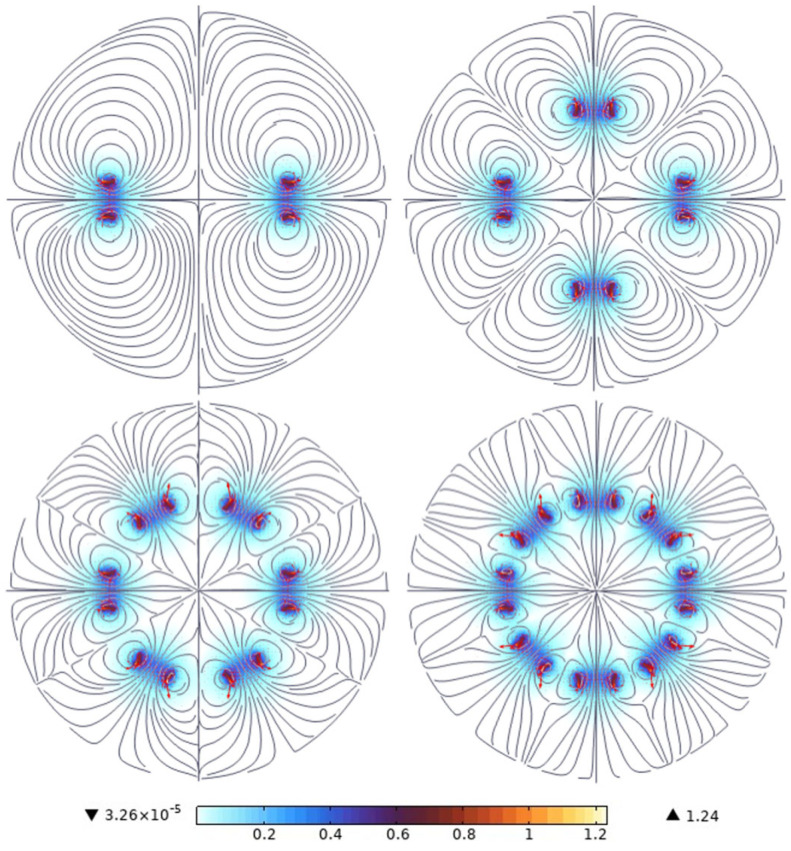
Magnetic field density distribution of different numbers of magnets.

Figure 3 illustrates the magnetic field intensity curves for different numbers of magnets. As the number of magnets increases, the distribution of the magnetic field intensity in space becomes more uniform, with a higher density observed at the edges of the magnets compared to their centers. The simulated magnetic field intensities for various magnet numbers are presented in Table 2. The results indicate that the average magnetic field intensity of eight magnets is the largest, which is 3.1 times that of two magnets. Therefore, at the same rotational speed, the rate of change for the magnetic field generated by eight magnets passing through the coil is higher, resulting in a larger alternating current (AC) output.

**Figure 4:**
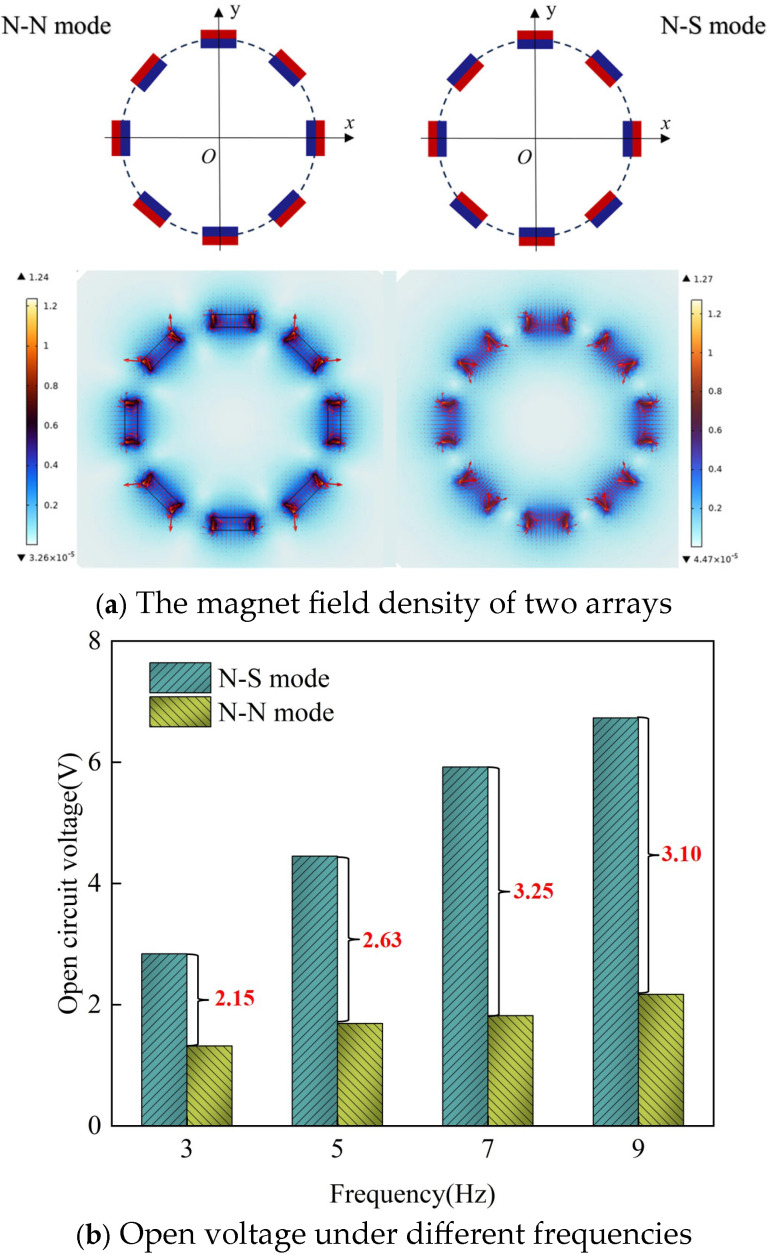
Performance comparison of two magnet arrays.

Figure 4 compares the magnet arrangements and output voltages under the same number of magnets. In the N-S mode, the magnet array features alternating N and S poles in the radial direction, whereas in the N-N mode, all N poles are aligned in the same direction. As shown in Figure 4a, the magnetic field density in the N-S mode is stronger than in the N-N mode. This indicates that when the N-S mode rotates, the magnetic field density varies at a higher rate, resulting in greater voltage output. According to the law of electromagnetic induction, the induced voltage depends on the ratio of the magnetic induction line and the magnetic flux [33]. Figure 4b illustrates the variation of open-circuit voltage for both magnet arrays at 3 Hz, 5 Hz, 7 Hz, and 9 Hz frequencies, respectively. As the frequency increases, the open-circuit voltage of the GEH increases. The open circuit voltage increases from 2.84 V to 6.73 V in N-S mode and from 1.32 V to 2.17 V in N-N mode, indicating that the N-S mode can generate higher voltage. Therefore, the magnetic field intensity of the N-S mode is higher than that of the N-N mode, and the magnetic flux change rate is larger, which makes the output performance of the N-S mode better.

### 3.2. Effects of Magnet Mass

**Figure 5:**
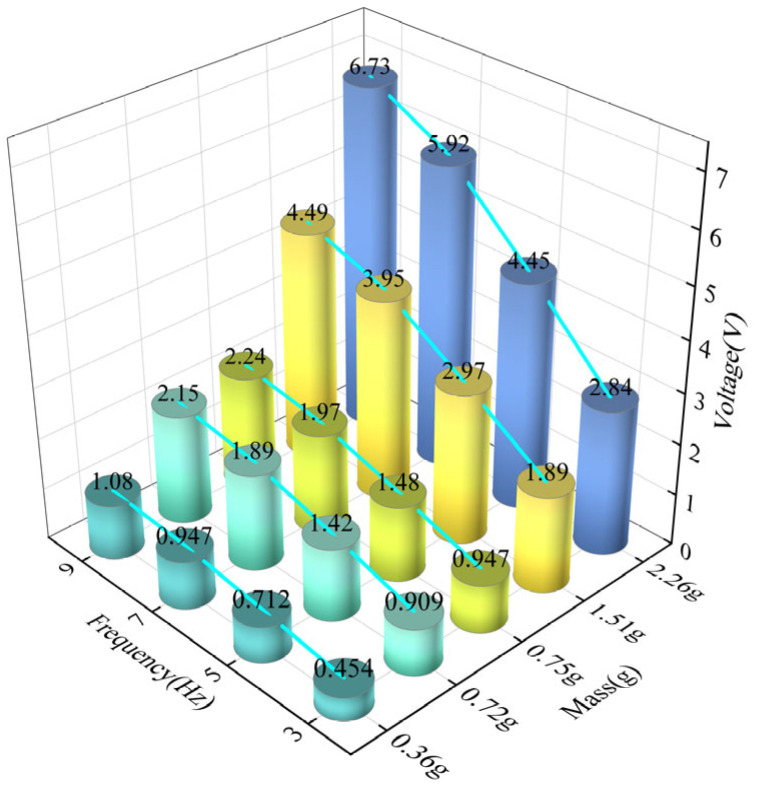
Influence of magnet mass on output voltage.

The N52 magnet type is selected to investigate the effect of varying magnet mass on output voltage. Figure 5 illustrates the effect of the N-S mode magnet mass of eight magnets on the output voltage of the GEH. Increasing the mass of the magnet, that is, increasing the volume of the magnet, increases the output voltage. The reason is that the change in the volume of the magnet will increase the magnetic field strength and magnetic field range, and the magnetic field will be more through the coil, and the induced voltage generated by the coil will be higher. When the magnetic field masses are close together, such as 0.72 g and 0.75 g, the GEH output voltage is basically equal, and the mass influence is less. Therefore, in a limited space, increasing the mass of the magnet will increase the magnetic field strength, which is conducive to improving the energy output of the energy harvester.

### 3.3. Determination of Coil and Magnet Distance

**Figure 6:**
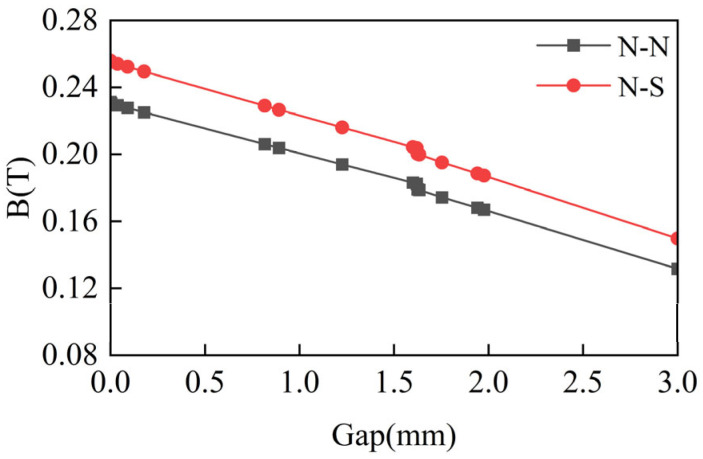
The magnetic field strength between coil and magnet under different gaps.

Magnetic field strength attenuates with the increase in distance; a reasonable choice of magnet and coil distance is conducive to enhancing the efficiency of the electromagnetic harvester. Figure 6 shows the magnetic field strength changes with distance. As can be observed, the magnetic field strength decreases with the increasing distance, which means that in practical applications, the closer the distance between the magnet and the coil, the more electrical energy. Considering the manufacturing error and assembly problem of the device, the distance is set to 2.0 mm. Therefore, the structural parameters of GEH were determined by the optimization scheme as shown in Table 3.

## 4. Electrical Performance Test

**Figure 7:**
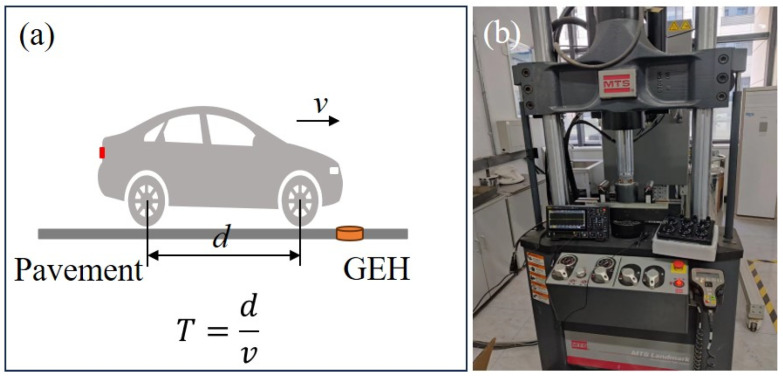
(**a**) Loading process of car on GEH; (**b**) photograph of the GEH on the MTS.

For the road energy harvesting technology, the electrical performance output is the key index for evaluating the energy harvester. Considering the actual service environment of the road, the time (*T*) for the vehicle’s front and rear tires (axle distance *d*) to pass through the device is selected as the reference index (Figure 7a), and the electrical performance of the optimized harvester was further evaluated by converting the vehicle driving speed at 30~100 km/h to a frequency of about 3 Hz~10 Hz [34]. The GEH prototype was tested using the MTS system (MTS Industrial Systems (China) Co., Ltd., Shanghai, China) to simulate the road environment (Figure 7b). The frequencies in the MTS system test were set as 3 Hz, 5 Hz, 7 Hz, and 9 Hz, respectively, and the maximum load displacement was 3.0 mm. A digital oscilloscope (DHO 924S, RIGOL, RIGOL Technologies Co., Ltd., Suzhou, China) was employed to measure and record the output voltage. Due to fluctuations in output voltage, the average value of the measured data was considered as the final result.

### 4.1. Voltage

#### 4.1.1. Open-Circuit Voltage

**Figure 8:**
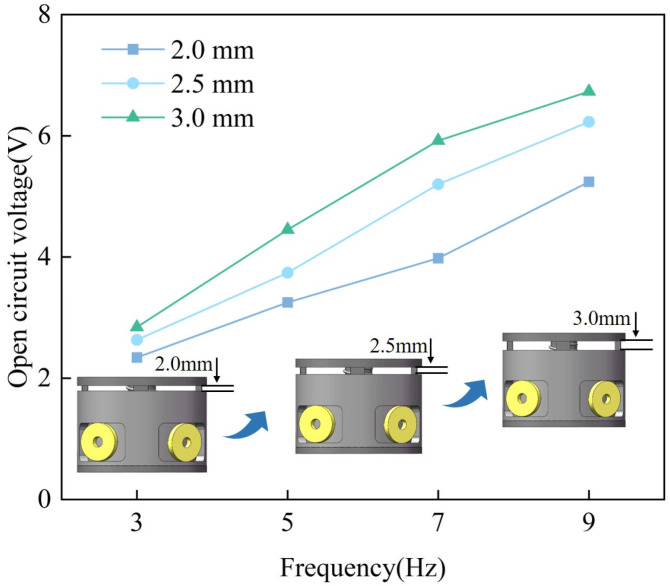
Open-circuit voltage of the GEH at different displacement and frequency.

Figure 8 shows the open-circuit voltage of the GEH at different displacements and frequencies. Under the same frequency, the open-circuit voltage increases with the increase in displacement. It is worth noting that with the same displacement, the open circuit voltage increases linearly with the increase in frequency. Compare open circuit voltages at different frequencies with a 3.0 mm displacement, the open circuit voltage of the harvester at 9 Hz reaches 6.73 V, which is 2.36 times that at 3 Hz. The results indicate that the output performance of the harvester is better at a higher frequency.

#### 4.1.2. Output Voltage

**Figure 9:**
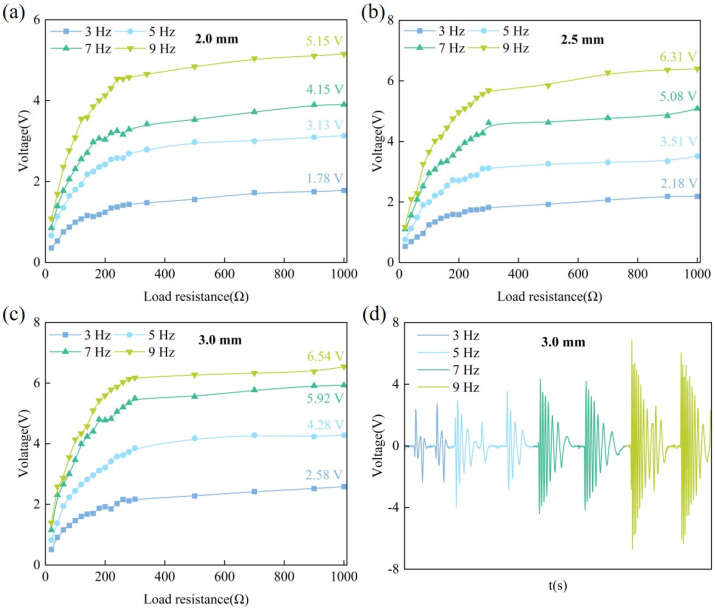
The output voltages of harvesters under different displacement and different frequencies: (**a**) 2.0 mm; (**b**) 2.5 mm; (**c**) 3.0 mm; (**d**) the voltage curve of the GEH at 3.0 mm displacement.

The effect of load resistance on output voltage at different frequencies is shown in Figure 9. Figure 9a–c depicts the relationship between output voltage and displacement for load resistance values ranging from 20 Ω to 1000 Ω.

As the load resistance increases, the output voltage of the GEH increases rapidly at first, and then becomes stable. Because the resistors and GEH are connected in series, the voltage value assigned to the device is proportional to its resistance value, and when the load resistance value is much higher than the internal resistance of the coil, the voltage value at the load is close to the total voltage of the circuit.

At the same frequency, the maximum output voltage rises with increasing displacement. At a frequency of 9 Hz, the open-circuit voltage of the energy harvester with a displacement of 2.0 mm is 5.15 V. As displacement increases, the output voltage rises by 22.52% (6.31 V) and 26.99% (6.54 V), respectively. Similarly, at a constant displacement, the output voltage gradually increases with frequency. For example, when the displacement is 3.0 mm, the frequency increases from 3 Hz to 9 Hz, and the corresponding output voltage increases by 65.89%, 129.45%, and 153.47%, respectively. Therefore, increasing the displacement and frequency is helpful to increase the output voltage of the GEH, but as load resistance increases, the output voltage increases first and then levels off. When the working voltage of the connected external circuit equipment is higher, the displacement can be appropriately increased or applied to the high-speed road to meet the output voltage needs of the equipment.

Figure 9d presents the output voltage of the prototype with different frequencies at 3.0 mm displacement. As the excitation frequency increases, both the waveform and amplitude of the output voltage rise, and the overall voltage output exhibits a damped vibration waveform. This phenomenon occurs because, during the rolling of the GEH by vehicle tires, the cover plate moves downward and subsequently resets upward. Under the interaction of the ratchet and pawl mechanism, the vertical motion of the cover plate is converted into unidirectional rotational motion. Once the pawl disengages from the ratchet, the ratchet continues to rotate by virtue of inertia. Through structural up-frequency conversion, low-frequency input is thus converted into high-frequency motion. Because the excitation frequency is relatively high, the mechanical inertia of ratchets made from different materials exerts only a minor influence on the output performance [35]. Therefore, this study does not separately investigate the effect of ratchet materials. Notably, at frequencies above 3 Hz, under a single excitation, the waveform is more compact and the working time is also increased, which significantly reflects the effect of up-frequency.

### 4.2. Output Power

**Figure 10:**
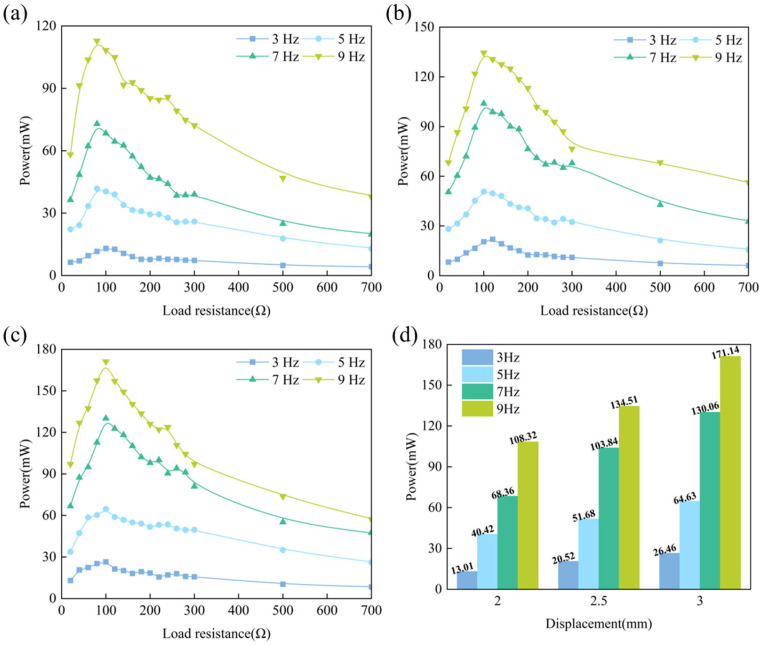
Output power of the GEH under different displacement and frequencies: (**a**) 2.0 mm; (**b**) 2.5 mm; (**c**) 3.0 mm. (**d**) Comparison of output power under different displacement.

To further evaluate the output performance, the output power under different load test conditions is depicted in Figure 10. The output power of the GEH shows a similar variation trend under different test conditions. With the increase in the external load resistance, the output power initially rises rapidly before subsequently decreasing. At a constant frequency, the output power increases linearly with the increase in displacement. At 9 Hz, the maximum power output reaches 171.14 mW at 3.0 mm displacement, 108.32 mW at 2.0 mm displacement, and 134.51 mW at 2.5 mm displacement. A displacement change of 1 mm will increase the output power by 1.58 times. Because under the larger vertical excitation the downward displacement of the screw bar is greater, the ratchet rotates faster and the flux change rate through the coil is greater, resulting in higher voltage and more power. When the displacement is fixed, the maximum output power increases significantly with the increase in frequency. For example, at 3 mm displacement, the maximum output power of the energy harvester at 9 Hz is 6.46 times that at 3 Hz. It is observed that under various conditions, the GEH achieves a peak output power at a resistance of approximately 100 Ω. Since the four coils are used in series, the total resistance of the four coils is 96 Ω. This is also consistent with the result obtained from Equation (7), that is, when *R_e_* equals *R_coil_* (96 Ω), the output power peaks. Therefore, when the GEH is connected to the external load, optimizing resistance matching ensures maximum output performance and enhances electromechanical conversion efficiency.

### 4.3. Output Power Density

Table 4 presents a comparison of the electrical performance of the GEH with that used or likely to be used for road energy harvesting. As the mechanical–electrical conversion principle, structure type, excitation condition, and size of current road energy harvester research are different, the voltage, power, and power density were used as evaluation indices to illustrate the comprehensive performance of this research.

According to Table 4, the maximum power density of the GEH at 9 Hz is 1277.16 mW/cm³, with an energy output per unit magnet mass of 70.63 mW/(cm³·g). The next highest power density, 98.18 W/m³ from [29], is over 13 times lower. Compared to other methods, traditional electromagnetic harvesters ([22,35]) show much lower power densities, while piezoelectric ([31,36]) and hybrid approaches ([37]) provide moderate improvements but still fall short. The minimum vertical displacement of the GEH is only 3.0 mm. Its mechanical components mainly achieve energy conversion through the coordination of the ratchet and pawl, which reduces the coordination of complex components and ensures high power output under driving comfort conditions. At the same time, the overall size of the GEH is significantly reduced, the structure is more compact, and the power density is higher. In short, the compact structure and simplified mechanical components of the GEH can minimize damage to pavement structure, enhance the long-term reliability of the energy harvester, and enable its adaptation to complex traffic environments.

**Table 4:** Comparison of the GEH with previous designs.

Reference	Energy Harvesting Method	Area or Volume	Frequency	Voltage (V)	Power (W)	Power Density
[38]	Electromagnetic	0.12 m^2^	1~2 Hz	-	3.21 × 10^−3^	0.027 W/m^2^
[22]	Electromagnetic	1.125 m^2^	0.5~2 Hz	0.13	3.21 × 10^−3^	0.0028 W/m^2^
[24]	Electromagnetic	-	1~5 mm/s	6.93	-	-
[32]	Piezoelectric	4.5 × 10^−5^ m^3^	80 km/h	61.0	0.62	13.80 W/m^3^
[30]	Electromagnetic	5.5 × 10^−3^ m^3^	6 km/h	51.2	0.54	98.18 W/m^3^
[39]	Electromagnetic–triboelectric	4 × 10^−4^ m^3^	0.1~0.5 Hz	7.0	-	50.81 W/m^3^
[36]	Piezoelectric	2.83 × 10^−4^ m^3^	4 Hz	7.48	5.19 × 10^−5^	0.18 W/m^3^
[37]	Piezoelectric	1.2 × 10^−2^ m^3^	13 Hz	80.0	0.52	43.33 W/m^3^
[40]	Piezoelectric–electromagnetic	2.3 × 10^−3^ m^3^	10 Hz	31.2	0.010	4.53 W/m^3^
This work	Electromagnetic	1.34 × 10^−4^ m^3^	9 Hz	6.73	0.17	1277.16 W/m^3^

### 4.4. Long-Lasting Stability

The application scenario of the GEH is focused on road traffic, where it must repeatedly withstand the rolling pressure of vehicle tires. Ensuring good structural stability and electrical output under long-term loading conditions is fundamental for the practical implementation of the GEH. Therefore, in this section, an MTS system was used to perform 100,000 loading cycles with a 3 mm displacement and 9 Hz frequency to test the variation in GEH output voltage and observe the device’s stability.

**Figure 11:**
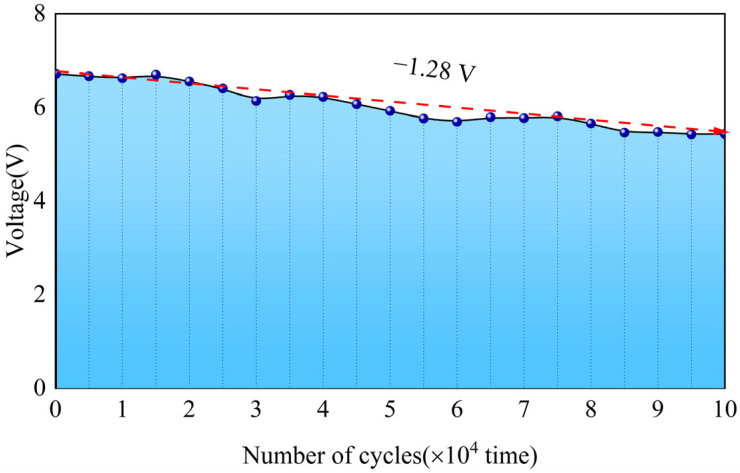
GEH output voltage in fatigue testing. The blue dot indicates 10,000 loads, and the red dashed line indicates the voltage drop trend.

Figure 11 illustrates the voltage output variation of GEH during 100,000 cycles. As the number of cycles increases, the harvester’s output voltage gradually decreases with some fluctuations, showing a minor decline of only 1.28 V compared to the initial voltage. Throughout the loading process, the harvester’s structure remained stable, with no cracks or damage observed. GEH exhibits excellent durability and electrical stability under long-term cycling, making it well-suited for complex road environments.

### 4.5. Actual Verification and Planning

**Figure 12:**
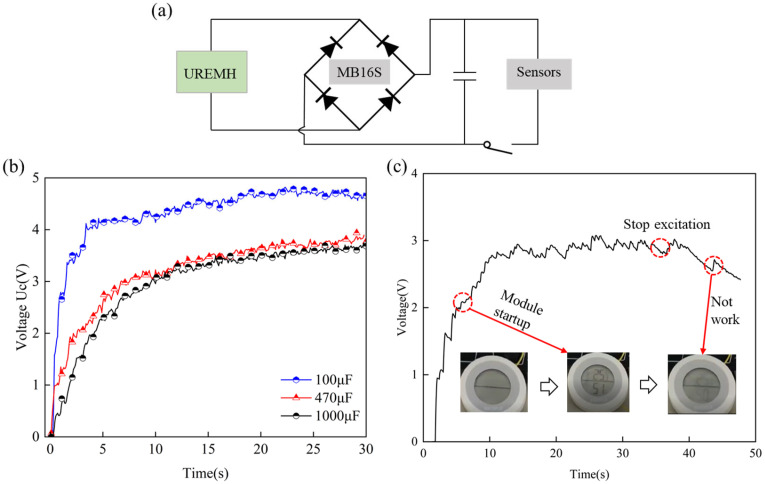
Energy harvester performance application test: (**a**) schematic diagram of circuit connections; (**b**) charging voltage on different capacitances; (**c**) charging the hygrothermograph.

To demonstrate the potential of the GEH as a sustainable power source for pavement applications, two sets of practical experiments were conducted in the laboratory. As shown in Figure 12a, the GEH output is rectified using a bridge rectifier and connected to a low-power device. The charging performance of different capacitors under test conditions 5 Hz–3.0 mm was shown in Figure 12b, where capacitors with values of 100 μF, 470 μF, and 1000 μF were charged to 4.65 V, 3.77 V, and 3.60 V within 30 s. The capacitance value increases while the charging voltage decreases. According to *E* = 0.5 *CU^2^*, the total energy output of the GEH remains constant, meaning that voltage is inversely proportional to capacitance [41]. It should be noted that the voltage fluctuations of the three capacitors is large, which will cause instability of the output power supply. In order to reduce the fluctuation, hybrid supercapacitors can be employed as energy storage components [42]. In Figure 12c, using the hygrometer as the load device under vibration excitation of 5 Hz, the output power of the GEH meets the hygrometer operation for a period of time. This demonstrates that the GEH has the potential to power various sensors in smart road applications, providing a viable alternative to lithium-ion batteries by addressing their lifespan limitations and the inconvenience of battery replacement. Consequently, it contributes to the development of low-carbon and sustainable road infrastructure.

**Figure 13:**
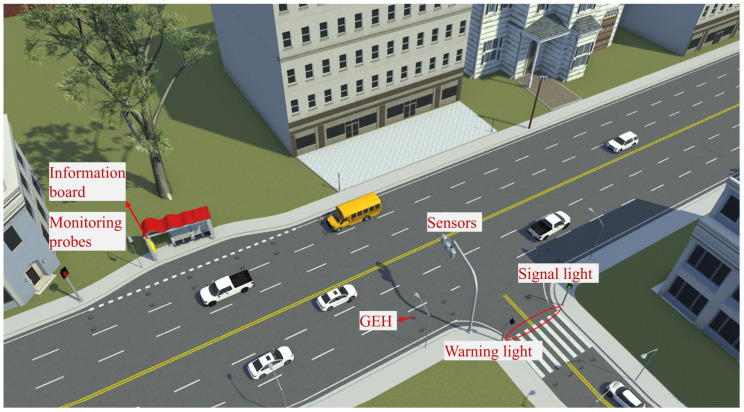
Application scenario of GEH on the road.

Figure 13 illustrates the application scenario of the GEH in smart roads. In response to the power supply requirements of smart roads, the energy output from the GEH can be utilized in two aspects. Firstly, it can power intelligent road sensors and nodes, such as pressure sensors and environmental monitoring sensors. Secondly, it can supply power to intelligent road warning systems, such as road contour guidance lights and LED warning lights. When a vehicle passes over the GEH, up-frequency and unidirectional rotation convert mechanical energy into electrical energy, which is then rectified and stored in supercapacitors or lithium-ion batteries to power the devices. Therefore, as the GEH is acted upon by the vehicle, it generates electricity, then charges sensors to monitor the road environment, or activates warning facilities to provide perception and early warning to the vehicle. Additionally, the real-time power supply from the GEH reduces dependence on the power grid, cuts transmission costs and maintenance fees, and promotes the green and sustainable development of smart roads.

## 5. Conclusions

In this study, a new GEH for pavement energy harvesting was introduced. The magnet arrays and the magnet–coil distance were optimized with the output performance as the index. Then, the GEH prototype was tested by MTS, and the output performance was analyzed in detail. The experimental data show that the designed energy harvester enables excellent performance. The following is a summary of the key results of this paper:

(1) The energy harvester can collect the road mechanical energy efficiently through the up-frequency and unidirectional rotation mechanism under the limited vertical displacement of 3.0 mm.
(2) The results show that the electrical performance of the GEH is improved when the frequency and displacement increase. Under 9 Hz excitation conditions, the output power of the GEH is 171.14 mW, the open circuit voltage is 6.73 V, and the output voltage drops by only 1.28 V after 100,000 consecutive loads.
(3) At a frequency of 5 Hz, the electronic hygrothermograph can work and the capacitor charge voltage exceeds 3.3 V, which proves the GEH has the prospect of power supply for low-power sensors.
(4) In the follow-up study, field tests in the actual road environment will be carried out to verify the working performance of the GEH, and research on the assembled structure will be carried out according to the sensor power supply requirements of the smart road to expand the application of the energy harvester.

## Figures and Tables

**Table 1:** Basic simulation parameters.

Magnet Size (mm)	Magnet Material	Number of Magnets	Residual Flux Density Model (mT)	Reverse Permeability	Air Relative Magnetic Permeability
10 × 10 × 3	N52	2~8	1.44	1.05	1

**Table 2:** Simulated values of magnetic density distribution under different number of magnets.

Number of Magnets	2	4	6	8
Average magnetic field intensity (mT)	18.3	35.6	48.2	57.5

**Table 3:** Geometric parameters of the GEH.

Parameters	Value
Material of magnets	NdFeB-N52
Number of magnets	8
Volume of magnets	10 × 10 × 3 mm^3^
Copper wire diameter	0.12 mm
Turns of each copper coil	500
Coil internal resistance	24 Ω
Gap between the coil and magnet	2 mm
Diameter of prototype	61 mm
Height of prototype	46 mm

## Data Availability

The original contributions presented in this study are included in the article. Further inquiries can be directed to the corresponding author.
